# The association between expression of *IFIT1* in podocytes of MRL/lpr mice and the renal pathological changes it causes: An animal study

**DOI:** 10.18632/oncotarget.13045

**Published:** 2016-11-03

**Authors:** Weiping Hu, Guodong Niu, Hongbo Li, Hanyuan Gao, Rudian Kang, Xiaoqing Chen, Ling Lin

**Affiliations:** ^1^ Rheumatism Department, The Second Affiliated Hospital of Fujian Medical University, Quanzhou, Fujian Province, China

**Keywords:** IFIT1, podocytes, animal study, renal pathological changes, lupus nephritis, Immunology and Microbiology Section, Immune response, Immunity

## Abstract

Renal damage is the major cause of SLE associated mortality, and *IFIT1*expression was elevated in SLE cases in accordance of previous studies. Therefore, we conducted an animal study to identify the role of *IFIT1* expression in renal pathological changes.18 female MRL/lpr mice and same number of female BALB/c mice were enrolled in present study. Quantitative analysis of urine protein, Complement C3 and C4, and anti-ds DNA antibody were conducted. HE and PAS staining and TEM analysis were employed to observe the pathological changes in renal tissue. Significant elevation on urine protein and anti-dsDNA and reduction on Complement C3 and C4 were observed in MRL/lpr mice when comparing the controls in same age. Staining and TEM analysis observed several pathological changes in glomerulus among MRL/lpr mice, including cellular enlargement, basement membrane thickening, and increased cellularcasts. The linear regression analysis found the optical density of *IFIT1* was inversely associated with F-actin, Nephrin, and Podocin, but not Synatopodin. In summary, *IFIT1* expression is associated with podocytes damage, and capable of suppressing some proteins essential to glomerular filtration.

## INTRODUCTION

Systemic lupus erythematosus (SLE) is a kind of common autoimmue disease which involves with multiple organ damage, and the incidence of SLE in global range is about 10-80 in 100,000. Lupus nephritis (LN) is the most frequently reported complication of SLE in clinical practice, about 50%-70% of SLE cases were diagnosed with LN, as the matter of fact, the pathological evidence showed almost all SLE cases developed renal impairment. Although great progress has been achieved in the medication treatment against SLE recently, however, about 10%-30% of SLE cases have advanced to end stage renal disease. Therefore, LN has become one of the major death causes of patients with SLE in recent years. Podocytes are epithelial cells that located in the outer layer of the glomerular basement membrane, which not only form the filtration barrier but also involve with the maintenance of glomerular capillaries and the repair process of basement membrane. In addition, podocytes also play critical role in the regulation of glomerular filtration and the immune response in human body. Growing evidence suggested that the damage of podocytes is the key of chronic renal pathological changes and both structure abnormal and malfunction of podocytes are of critical importance in the development of LN [1-2].

Nephrin is structural component of the slit diaphragm which is essential of maintaining proper function of renal filtration barrier, the defect on the expression and distribution of Nephrin could be the underlying mechanism of various glomerulopathy. The animal experiment also supported that the gene knockout of Nephrin or the administration of Nephrin antibody would result in the massive production of proteinuria [3]. Podocin is a necessary component of the cell membrane of podocytes, it can interact with Nephrin and CD2 associated protein and help to stabilize the podocytes. Moreover, it also has signaling function, such as enhancing the signal transmission of Neprhin. The absence of podocin in mice could lead to the abnormal slit diaphragm and eventually die of massive proteinuria [4]. As for F-actin, it is the fundamental element of podocytes movement, in the case of podocyte movement change, F-actin would rearrange and consequently the proteinuria occurs [5].

Several clinical studies indicated that the serum level of interferon alpha (*IFN-α*) were significantly higher in SLE patients when comparing with healthy group, and it also closely associated with some other indicators, such as the activity and severity of SLE and the anti-dsDNA antibody titer [6]. The knockout of *IFN* receptor in NZB/NZW F1 mice can lead to the significant reduction of antinuclear antibody, and also remission of autoimmune hemolytic anemia, consequently the mortality rate of mice would decrease [7]. Type I interferon signaling pathway is of critical importance in the production of anti-dsDNA antibody and the development of LN in the TMPD induced lupus mice, therefore, blocking this specific pathway would render protection on lupus prone mice [8]. The experimental data acquired from lupus mice also suggested that IFN-α can further aggravate the renal damage, especially targeted on the podocytes [9]. Evidence about the association on the organ damage and *IFN* also found in human, data suggested the expression of *IFN* inducible genes have elevated in SLE patients, and also associated with some clinical manifestation of lupus, and damage in kidney and nervous system [10].

Interferon-induced protein with tetratricopeptide repeats 1(*IFIT1*) is one of interferon inducible genes and normally its mRNA level is extremely low in intracellular environment, however, the mRNA level can be induced by interferon and other viruses. Under such circumstance, the mRNA level can be elevated to about 100-fold higher than other interferon inducible genes, which ranked as the top among all genes induced by type I interferon [11]. It has been proved that the expression of *IFIT1* was elevated in the peripheral blood samples from SLE patients, and similarly same elevation was observed in renal tissue of LN patients [12]. Podocytes can produce massive *IFIT1* with the stimulation of interferon or virus. However, the role of up-regulated expression of *IFIT1* in the podocytes pathological changes in LN patients has not yet been identified. Therefore, we conducted an animal experiment to clarify these above mentioned problems.

## RESULTS

### Urine protein

As can be seen in Table 1, the mean urine protein ranged from 0.99 mg/L to 1.04 mg/L in BALB/c mice groups, and 2.11 mg/L to 6.42 mg/L in MRL/lpr mice groups. According to the results of statistical analysis, no significant difference observed when comparing the urine protein within BALB/c mice by month of age (*P* = 0.934), suggesting no renal function difference was found in control groups. But in comparison performed among different groups in same age, statistical results suggested that the urine protein in MRL/lpr mice was significantly higher(3M *versus* 3B, *P* < 0.001; 4M *versus* 4B, *P* < 0.001; 5M *versus* 5B, *P* < 0.001). Within MRL/lpr mice groups, the urine protein was elevated with the growth of age, and statistical significance was also observed (*P* < 0.001).

**Table 1:** The comparison of urine protein, C3, C4 and anti-dsDNA level between MRL/lpr mice and BALC mice

Animal group (n=18)	Age subgroup (n=6)	Urine protein (mg/L)	C3 (ug/L)	C4 (ug/L)	Anti-dsDNA (pg/L)
BALB/c	3 months	1.01±0.10	98.86±15.92	26.23±4.58	37.65±1.75
	4 months	1.04±0.30	103.50±16.55	28.76±2.60	38.62±3.17
	5 months	0.99±0.24	101.73±18.93	28.76±3.51	37.05±2.32
MRL/lpr	3 months	2.11±0.29*^Δ^	86.39±5.46^Δ^	17.66±1.07*^Δ^	125.93±7.50*^Δ^
	4 months	3.56±0.26*^Δ^	78.42±3.26*^Δ^	15.31±0.47*^Δ^	185.79±8.95*^Δ^
	5 months	6.42±0.57*^Δ^	57.09±1.82*^Δ^	13.73±1.04*^Δ^	208.39±21.16*^Δ^

* *P*<0.05 compared with same age of control

Δ *P*<0.05 compared within MRL/lpr mice by One-way ANOVA

### Complement components and anti-ds DNA antibody

Table 1 also reports the data on Complement C3 and C4, and anti-ds DNA antibody for all animal subjects. The mean Complement C3 ranged from 98.86 ug/L to 103.50 ug/L in BALB/c mice, and 57.09 ug/L to 86.39 ug/L in MRL/lpr mice. The results revealed that the components C3 level was not significantly different between 3 months BALB/c mice and MRL/lpr mice (3B *versus* 3M, *P* = 0.10), but the significant reduction in component 3 level was observed among 4 and 5 months of MRL/lpr mice when comparing same age of BALB/c mice(4B *versus* 4M, *P* = 0.005; 5B *versus* 5M, *P* < 0.001). According to the results of one-way ANOVA performed among MRL/lpr mice, significant decrease in Complement C3 was evident between groups assigned by age(*P* < 0.001). As for Complement C4, strong evidence of significant reduction was found in MRL/lpr mice when we compare with BALB/c mice in same age (3B *versus* 3M, *P* = 0.001; 4B *versus* 4M, *P* < 0.001; 5B *versus* 5M, *P* < 0.001). Similar with the results of one-way ANOVA performed in components C3, we found evidence of significant reduction within MRL/lpr mice (*P* < 0.001). The mean anti-ds DNA antibody ranged from 37.05 pg/L to 38.62 pg/L in BALB/c mice, and ranged from 125.93 pg/L to 208.39 pg/L in MRL/lpr mice. The *P* values of Student's *T*-test were all smaller than 0.001 when comparing the anti-ds DNA antibody between BALB/c mice and MRL/lpr mice in same age. In addition, we also found that the anti-ds DNA antibody significantly increased with the month of age in MRL/lpr mice (*P* < 0.001).

### Renal pathological analysis

**Figure 1:**
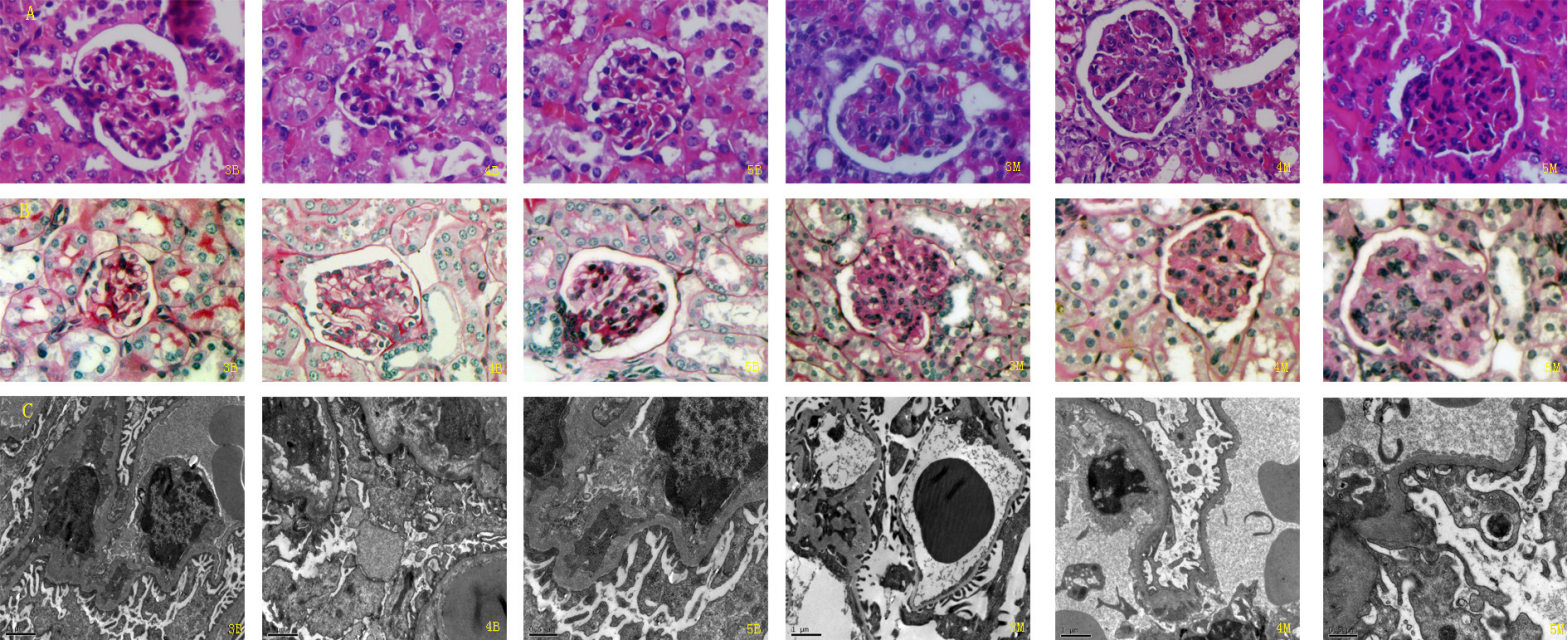
The renal pathological changes in animal subjects A. HE staining; B. PAS staining; C. TEM analysis (original magnification ×10000)

We attempted to investigate the renal pathological changes by using multiple methods, including HE and PAS staining, and TEM analysis. From Figure 1 we can see that the cell count in glomerulus was significantly increased among MRL/lpr mice when comparing with their counterparts in same age after HE staining. Moreover, the proliferation of mesangial cells was enhanced and basement membrane thickening was also observed. PAS staining revealed several pathological changes in glomerulus among MRL/lpr mice, including cellular enlargement, basement membrane thickening, and increased cellularcasts. These above mentioned pathological changes demonstrated an aggravated trend with the growth of age among MRL/lpr mice based on the data we collected in this study. By using transmission electron microscope, podocyte foot process effacement and expanded width were detected among MRL/lpr mice when comparing with same age of BALB/c mice. Partial basement membrane thickening was also observed under the original magnification of×10000, and the pathological change of thickening was progressed with age of month among MRL/lpr mice(See Figure 1).

### IFIT1 expression and other podocytes proteins

We employed ImageJ2X software to calculate the optical density from images created by immunofluorescence microscopy (Supplementary Figure S1-S4), and data was presented in the form of mean±SD(See Table 2). The first set of analyses examined the difference on four podocytes proteins and *IFIT1* expression between BALB/c mice and MRL/lpr mice, and the effect of age on the protein level within MRL/lpr mice. Among four podocytes proteins we investigated, all demonstrated significant reduction in MRL/lpr mice when comparing with same age of BALB/c mice (*P* < 0.05). Similarly, we also observed significant decrease within MRL/lpr mice as the age growth in accordance with the results of one-way ANOVA(*P* < 0.05). However, no significant difference was found between *IFIT1* expression between BALB/c mice and MRL/lpr mice in age of 3 months (*P* = 0.237). As for the rest of subjects, significant elevation in *IFIT1* expression level was observed in MRL/lpr mice (4B *versus* 4M, *P* = 0.019; 5B *versus* 5M, *P* = 0.005). Within the MRL/lpr mice, the difference between ages was also found in accordance with the results of one-way ANOVA(*P* < 0.001).

**Table 2:** The comparison of podocytes proteins and IFIT1 expression level between MRL/lpr mice and BALC mice

Variables	Animal group (n=18)	Age subgroup (n=6)	Optical density
F_actin	BALB/c	3 months	0.13±0.11
		4 months	0.13±0.01
		5 months	0.14±0.01
	MRL/lpr	3 months	0.10±0.01*^Δ^
		4 months	0.08±0.01*^Δ^
		5 months	0.07±0.01*^Δ^
Nephrin	BALB/c	3 months	0.14±0.10
		4 months	0.15±0.11
		5 months	0.14±0.01
	MRL/lpr	3 months	0.12±0.01*^Δ^
		4 months	0.10±0.01*^Δ^
		5 months	0.09±0.01*^Δ^
Podocin	BALB/c	3 months	0.13±0.02
		4 months	0.14±0.01
		5 months	0.13±0.02
	MRL/lpr	3 months	0.10±0.01*^Δ^
		4 months	0.09±0.01*^Δ^
		5 months	0.07±0.01*^Δ^
Synatopodin	BALB/c	3 months	0.14±0.01
		4 months	0.13±0.01
		5 months	0.13±0.17
	MRL/lpr	3 months	0.12±0.01*^Δ^
		4 months	0.10±0.01*^Δ^
		5 months	0.08±0.01*^Δ^
IFIT1	BALB/c	3 months	0.14±0.01
		4 months	0.13±0.01
		5 months	0.12±0.01
	MRL/lpr	3 months	0.15±0.01*^Δ^
		4 months	0.14±0.01*^Δ^
		5 months	0.15±0.02*^Δ^

* *P*<0.05 compared with same age of control

Δ *P*<0.05 compared within MRL/lpr mice by One-way ANOVA

In order to assess the correlation between four podocytes proteins and *IFIT1* expression in animal subjects, we further conducted linear regression analysis to estimate the coefficients. Table 3 displays the summary statistics of linear regression analysis in all animal subjects. As can be seen, F-actin, Nephrin, and Podocin were inversely associated with the expression of *IFIT1* in animal renal tissue(*P* < 0.05). However, no statistically significant correlation between Synatopodin and *IFIT1* expression was found (*P* = 0.051).

**Table 3:** Simple linear regression analyses: predicted influence of four podocytes proteins on IFIT1 expression

Variables	Coefficient(SE)	Standardized coefficient	t	*P*-value
F-actin	−0.318(0.088)	−0.528	−3.629	0.001*
Nephrin	−0.359(0.108)	−0.495	−3.326	0.002*
Podocin	−0.299(0.089)	−0.498	−3.348	0.002*
Synatopodin	−0.224(0.111)	−0.328	−2.026	0.051

* *P*<0.05

## DISCUSSION

MRL/lpr mice model was firstly established by Murphy and Roths in 1978 by using multiple inbred mice, and this kind of mice have a life span of 5 to 7 months. They can be utilized as a study tool for investigating SLE because they would develop a systemic autoimmune disease similar to human SLE, characterized by antibody production, immunocomplex formation and fatal nephritis [13]. In detail, the mice would begin to manifest pathological changes in age of 12 weeks, and the vasculitis and renal pathological changes occurred approximately between age of 15 to 20 weeks. The renal pathological changes in MRL/lpr mice are very similar with human LN, for example, inflammatory cell infiltration, vasculitis, endocapillary or extracapillary proliferative lesions and tubuloreticular inclusion. Therefore, such characters enable MRL/lpr mice as an ideal animal model to be used in investigation of SLE.

The complement deficiency is a common clinical manifestation among patients with SLE, and has been verified by some prospective studies [14-15]. The pathogenesis role of hypocomplementemia in SLE development has not yet been fully determined, but prior studies have indicated that the massive immune complexes induced by the progress of SLE would activate complement system, and consequently lead to the deposition of complement in serum [16]. Moreover, complement deficiency may also associated with renal damage among patients with SLE, some prospective studies observed the inverse association between complement level and the renal function, suggesting reduction on complement not only linked with the disease activity but also renders negative effects on kidney [17-18]. Consistent with previous findings, we observed the significant reduction on both Complement C3 and C4 among MRL/lpr mice, and elevated urine protein in these subjects, while no such pathological changes detected among BALB/c mice. With regard to the comparison on anti-ds DNA antibody level between MRL/lpr mice and controls, we also found that this specific indicator of SLE was significantly elevated in MRL/lpr mice, suggesting the animal models we employed in this study were solid and robust.

The main purpose of this study was to identify the association between *IFIT1* expression and renal pathological changes, therefore, we applied multiple methods to examine the renal tissue obtained from mice, including staining, TEM analysis and immunofluorescence microscopy. The most striking finding is that we observed the inverse association between *IFIT1* expression and four podocytes proteins except synsptopodin which are essential in maintaining normal function of glomerular filtration. Although the *P* value of correlation between *IFIT1* expression and Synsptopodin was marginal, still we found that Synsptopodin was significantly reduced as disease progressed among MRL/lpr mice. We assume that it may attribute to the sample size. Therefore, we can assume that the up-regulated *IFIT1* expression is associated with the impairment of podocytes and may has potential to trigger LN in mice. The pathological analysis further corroborated the assumption, due to the massive loss of podocytes in renal tissue of MRL/lpr mice and the compromised renal function were also identified as the *IFIT1* expression elevates. Currently, *IFIT1* is generally acknowledged as an important gene which carries both antivirus property and immune regulation. Data from several sources have pointed out that *IFIT1* gene is capable of interacting with eIF3 which subsequently suppresses more than 60% of translation in cell and viruses during protein synthesis [19]. The absence of *IFIT1* gene may lead to the enhancement of virus replication, and gene knockout performed by using RNA interference in human hepatocytes can directly increase the HCV RNA titer [20]. Up to now, there is little published information on the underlying mechanism of *IFIT1* expression and SLE occurrence and progression. A possible explanation for the pathogenesis of *IFIT1* might be the ability of inducing the production of both *IL-4* and *IL-10* [21], and consequently cause Th1/Th2 imbalance which is commonly observed in SLE patients. Latest research involving sixty SLE patients found that, comparing with healthy volunteers, the *IL-10* production was significantly elevated in SLE cases, and also positively correlated with disease activity [22]. In addition, SLE patients are prone to virus infection due to the abnormality in immune response when comparing with healthy counterparts, the vulnerability may induce the expression of *IFIT1*. Although the mechanism between *IFIT1* expression and podocytes damage has not yet been identified, we did observe the inverse association between the *IFIT1* expression and some podocytes proteins which are essential to maintain normal function of glomerular filtration. This can partly explain the association between *IFIT1* expression and renal pathological changes, suggesting *IFIT1* may involve in LN.

Although our study was limited by the sample size of animal subjects, it supported the potential role of *IFIT1* in renal pathological changes, as well as podocytes damage. To the best of our knowledge, it is the first report on the *IFIT1* and renal damage in animal model, and its unfavorable effect may work through destructing the cell structure of podocytes. We believe that further cell transfection and transcriptome analysis are warranted to identify the signal pathway of renal pathological changes caused by *IFIT1* expression and to provide evidence for the establishment of novel therapeutic strategy against SLE.

## MATERIALS AND METHODS

### Animals

18 female MRL/lpr mice and same number of female BALB/c mice served as control were supplied from Animal Center of Chinese Academy of Science. All mice were housed in air conditioned animal facility and subjected to 12h dark/light cycle. All animal experiments conducted were sanctioned by the Ethical Committee of Fujian Medical University. 18 female MRL/lpr mice were further divided into 3 groups by age with identical number (*n* = 6), namely 3 months, 4 months, and 5 months(marked as 3M-5M in all figures). Identical group assignment was applied in 18 female BALB/c mice(marked as 3B-5B in all figures).

### Urine protein test

24-hour urine collection was conducted at the same day of all animal subjects after 2 weeks of enrollment and then stored in −4°C refrigerator. Quantitative analysis of urine protein was performed by using cobas 6500 analyzer (Roche, Basel, Switzerland), and all procedures were followed the manual strictly.

### Enzyme-linked immunosorbent assays of serum sample

Blood sample was acquired from all animal subjects and centrifuged at 3000rpm for 20 min in room temperature to separate serum. Complement C3 and C4 and anti-double strand DNA antibody(Bioleaf Biotech, Shanghai, China) in serum were quantified by using enzyme-linked immunosorbent kit, and all procedures were conducted strictly in accordance with the manufacturers' instructions.

### Renal pathological change

Renal tissue was obtained from all animal subjects after sacrifice, then fixed with 10% formalin for 48 hours. Tissue was subjected to dehydration by graded ethanol treatment and then paraffin wax infiltration was performed. Finally, the tissue was cut into section of 4 to 7 um and was stained with hematoxylin and eosin (HE) reagent for pathological histological examination. Moreover, periodic acid-schiff (PAS) stain and transmission electron microscope (TEM) analysis were also performed by using JEM1230 electron microscope (JEOL, Tokyo, Japan).

### Immunofluorescencemicroscopy of F-actin, nephrin, podocin, synatopodin and IFIT1

The paraffin sections were placed on positively charged microscope slides and heated in tissue-drying over for 30 min at 65°C. After drying process, the slides were washed in xylene for two timesfor 15 min each at room temperature. Wash slides in graded ethanol for 5 minutes each and then rinse slides gently with running distilled water for 10 min. Boil slides in 0.01M sodium citrate buffer at 99-100°C for 15 min. Remove the slides from heat and cool down to room temperature, then rinse in 0.02M PBS for three times for 3 min each. Apply primary antibodies (Nephrinantibody, Biorbyt ORB16038, Cambridge, UK; Podocin antibody, Abcam AB50339, MA, USA; F-actin antibody, Abcam AB 205, MA, USA; Synsptopodin antibody, Abcam AB101883, MA, USA;*IFIT1* antibody, Santa Cruz SC-82945, TX, USA.) diluted in incubation buffer for overnight in 4°C, then rinse in 0.02M PBS for three times for 3 min each. Apply second antibodies diluted in incubation buffer and incubate in room temperature for 1 hour, then rinse in 0.02M PBS for three times for 3 min each. Add 1: 500diluted DAPI solution and store at −20°C, and visualize using a fluorescence microscope.

### Statistical analysis

All data acquired from this study were expressed in the form of means±SD. Statistical analysis was performed by SPSS version 22.0 (Chicago, IL, USA). One-way analysis of variance or Student's T test was employed to compare urine protein, complement C3 and C4, anti-double strand DNA antibody and optical density from immunofluorescence analysis. The correlation coefficient between *IFIT1* expression and podocytes proteins was estimated by using linear regression analysis. A *P* value less than 0.05 was considered significant.

## SUPPLEMENTARY MATERIALS FIGURES


