# Supplementary material for: The association between expression of IFIT1 in podocytes of MRL/lpr mice and the renal pathological changes it causes: An animal study
Source: Oncotarget. 2016 Nov 3;7(47):76464–70. doi: 10.18632/oncotarget.13045 (PMC5363523; doi:10.18632/oncotarget.13045)
Supplement: Supplementary file 1 [file oncotarget-07-76464-s001.pdf]

Expression of functional alternative telomerase RNA component gene in mouse brain and in motor neurons cells protects from oxidative stress

Supplementary Material

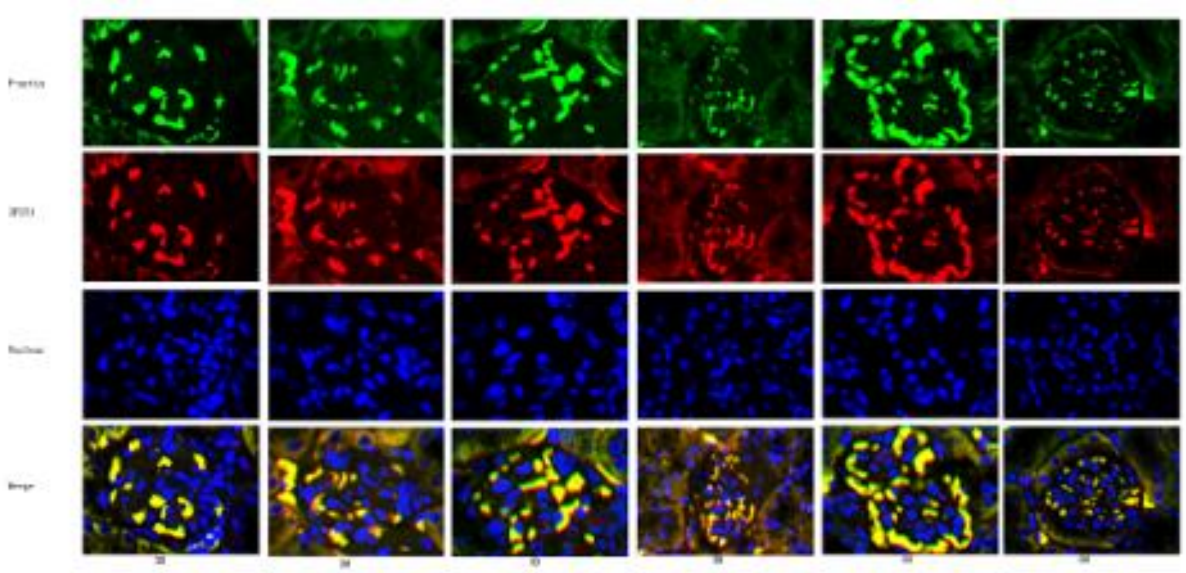

Figure S1: The immunofluorescence results of F-actin obtained after original magnification ×400

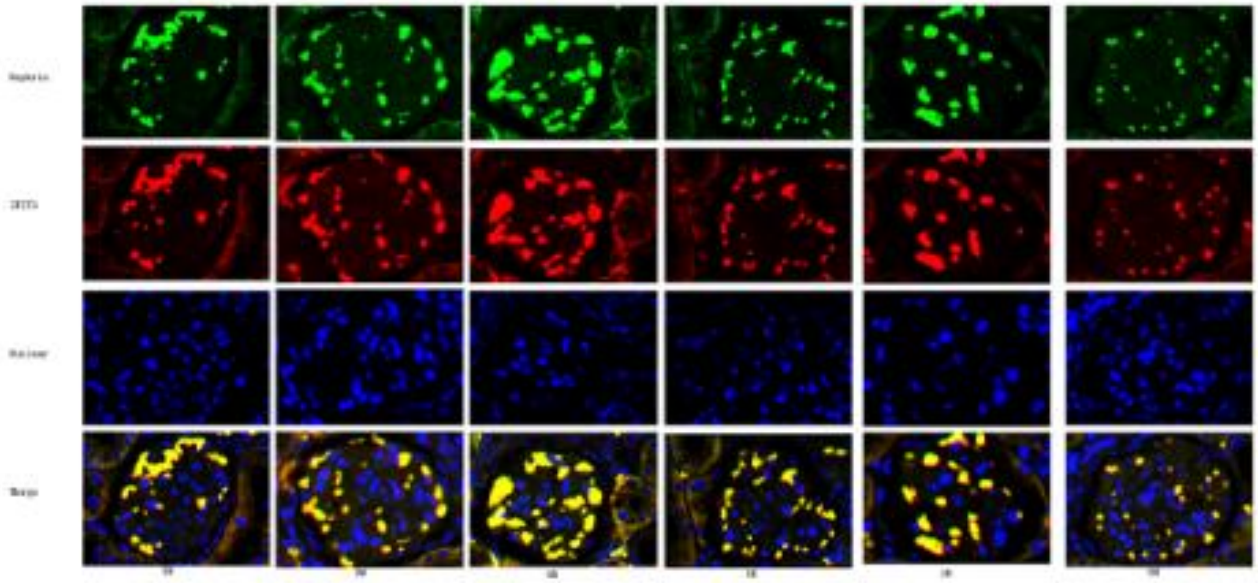

Figure S2: The immunofluorescence results of Nephrin obtained after original magnification ×400

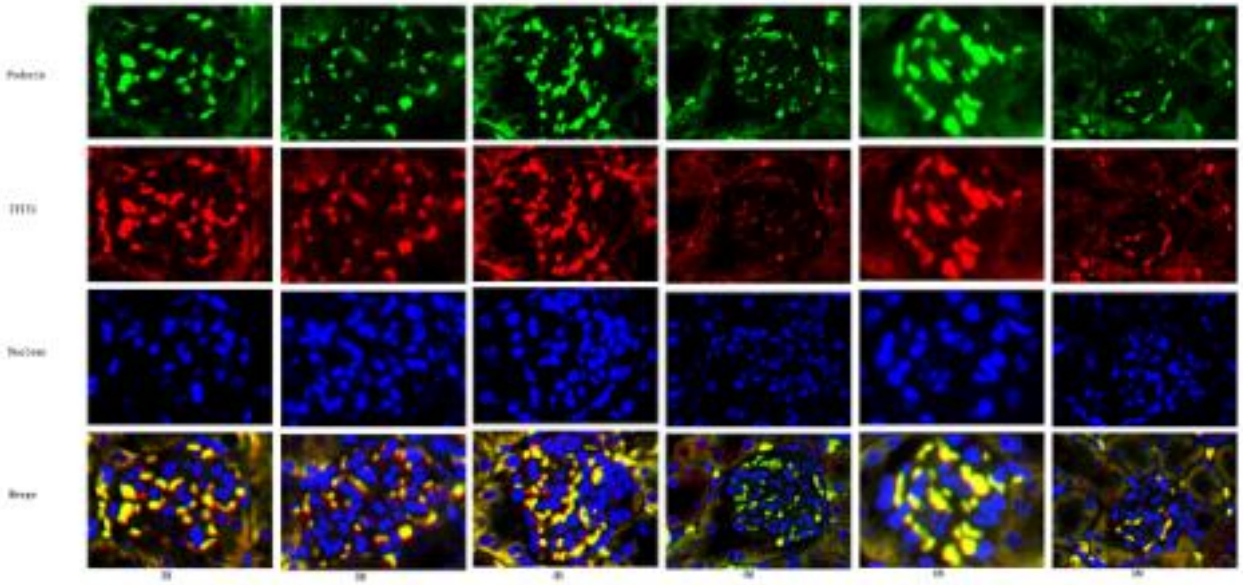

Figure S3: The immunofluorescence results of Podocin obtained after original magnification ×400

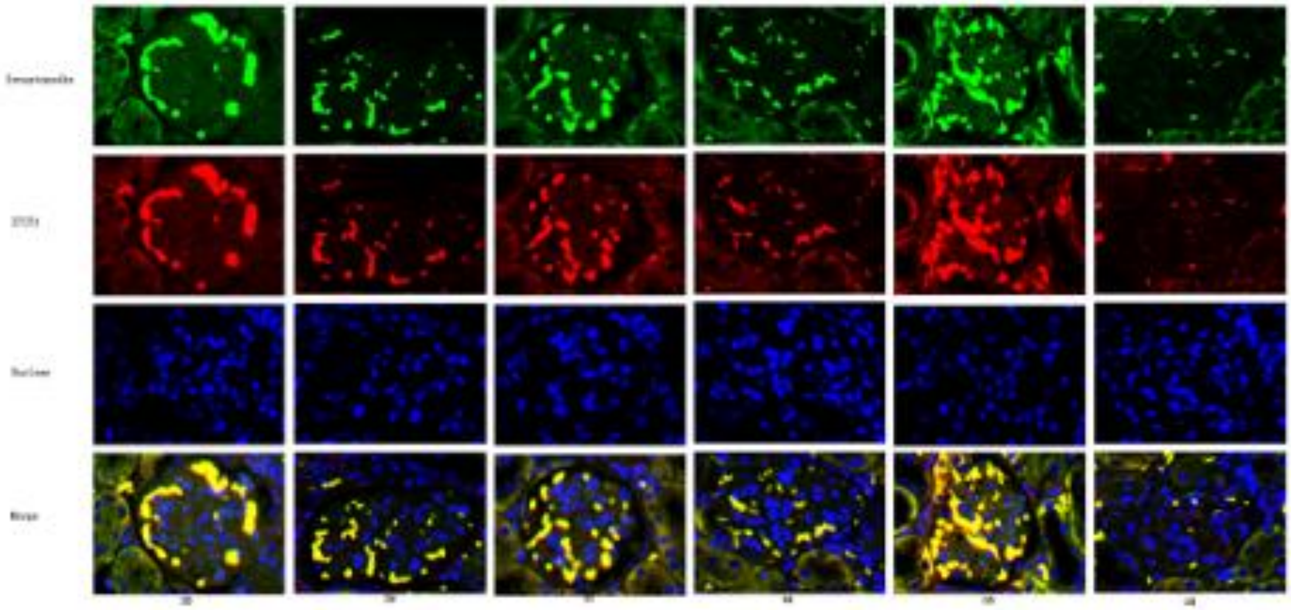

Figure S4: The immunofluorescence results of Synatopodin obtained after original magnification ×400
